# An examination of the benefits of health promotion programs for the national fire service

**DOI:** 10.1186/1471-2458-13-805

**Published:** 2013-09-05

**Authors:** Walker SC Poston, Christopher K Haddock, Sara A Jahnke, Nattinee Jitnarin, R Sue Day

**Affiliations:** 1 Center for Fire, Rescue and EMS Health Research, Institute for Biobehavioral Health Research, NDRI-MA, NDRI: National Development and Research Institutes, Inc, 1920 West 143rd Street Suite 120, Leawood, KS 66224, USA; 2 Division of Epidemiology, Human Genetics, and Environmental Sciences, Michael & Susan Dell Center for Healthy Living, University of Texas Houston Health Sciences Center, 1200 Herman Pressler, RAS Building, E1027, Houston TX 77030, USA

**Keywords:** Health promotion, Firefighter, Obesity, Fitness

## Abstract

**Background:**

Firefighters suffer from high prevalence of obesity, substandard fitness, and cardiovascular-related deaths. There have been a limited number of firefighter health promotion programs that have been developed and empirically-tested for this important occupational group. We evaluated the health of firefighters from departments with well-developed health promotion programs and compared them with those from departments not having such programs using a large national sample of career fire departments that varied in size and mission. We measured a broad array of important individual firefighter health outcomes (e.g., body composition, physical activity, and general and behavioral health) consistent with national fire service goals and addressed significant statistical limitations unaccounted for in previous studies.

**Methods:**

Using the approach of purposive sampling of heterogeneous instances, we selected and conducted a national evaluation of 10 departments already implementing wellness and fitness programs (Wellness Approach; WA) with 10 departments that did not (Standard). Participants were 1,002 male firefighters (WA n = 522; Standard n = 480) who underwent assessments including body composition, fitness, and general/behavioral health (e.g., injury, depressive symptoms).

**Results:**

Firefighters in WA departments were healthier than their Standard department counterparts. For example, they were less likely to be obese (adjusted [A]OR = 0.58; 95% CI = 0.41-0.82), more likely to meet endurance capacity standards for firefighting (AOR = 5.19; 95% CI = 2.49-10.83) and have higher estimated VO_2max_ (40.7 ± 0.6 vs. 37.5 ± 1.3 for firefighters in Standard departments; p = 0.001). In addition, WA firefighter were substantially less likely to smoke (AOR = 0.30; 95% CI = 0.17-0.54) or ever have been diagnosed with an anxiety disorder (AOR = 0.27; 95% CI = 0.14-0.52) and they expressed higher job satisfaction across several domains. However, WA firefighters were somewhat more likely to have reported an injury to Workers’ Compensation (AOR = 1.74; 95% CI = 1.05-2.90). It was notable that both groups evidenced high prevalence of smokeless tobacco use and binge drinking.

**Conclusions:**

Firefighters in departments selected based on having strong wellness programs (WA) were healthier along a number of dimensions important to firefighter wellness and operational readiness. However, several health areas require greater attention including problematic alcohol consumption and smokeless tobacco use, suggesting that more emphasis on these behavioral health issues is needed in the fire service.

## Background

The US Fire Service faces several daunting health crises including high prevalence of obesity, substandard fitness, and cardiovascular-related deaths [1]. Many career firefighters struggle with excessive adiposity and low fitness, with recent studies documenting prevalence estimates of overweight and obesity (BMI≥25.0 kg/m^2^) ranging from 73%- 88% [1-3]. Clinical obesity prevalence (BMI ≥ 30.0 kg/m^2^) among firefighters is between 30%-40%, similar to that found for US adults [4]. While some have questioned whether these estimates are artificially inflated because of misclassification of muscular firefighters with low body fat (but high BMIs), studies measuring body composition using multiple methods demonstrated that this concern is unfounded [3-5]. Firefighters also experience small, steady weight gain throughout their careers [6-8]. For example, several studies have documented average weight gains between 1.2-3.4 lbs/year [6-8].

Obesity also has a negative impact on productivity and healthcare costs among firefighters. Soteriades et al. [9] prospectively evaluated disability risk over more than six years and found each BMI unit increase was associated with a 5% increase in the likelihood of a firefighter becoming disabled. We examined the relationship between obesity and injury-related absenteeism and found that overweight and obese male firefighters missed 2.7-5.0 times (depending on their weight status category) the number of work days compared to normal weight peers [10]. The financial costs to departments per firefighter increased dramatically as firefighters were overweight ($74), Class I obese ($254), and Class II and III obese ($1,683) [10].

Substandard fitness also plagues the fire service. For example, Donovan and colleagues [11] reported that 25% of their sample of 214 male career firefighters failed to meet accepted standards for cardiorespiratory fitness (VO_2max_ = 42.0 ml/kg/minute) and that estimated VO_2max_ was lower in firefighters with metabolic syndrome. More recently, Poston et al. [3] reported that over 60% of their population-based sample of male career firefighters failed to meet the above criterion and that risk for not meeting the standard was significantly higher for obese firefighters when compared to their normal weight colleagues. Durand and associates [12] found that only 20% of their sample of career firefighters exercised regularly and met or exceeded recommendations for 150 minutes per week of moderate or greater exercise. In addition, they also found that greater physical activity was associated with better cardiovascular profiles (e.g., lower triglycerides and glucose levels) even after adjustment for body composition and smoking [12].

The epidemics of low fitness and obesity are critical issues facing the fire service because of their association with injury risk and lowered occupational readiness. Firefighter injuries are very costly and primarily due to overexertion during exercise and training drills and fireground and emergency medical operations [13,14]. Firefighters who exercised regularly were found to be at half the risk for non-exercise injuries, which typically represent over 60% of all firefighter injuries [15]. Relatedly, we also demonstrated that baseline weight status was a significant prospective predictor of incident musculoskeletal injuries, with obese (BMI ≥ 30 kg/m^2^) male firefighters being 5.2 times more likely (95% CI = 1.1-23.4) to experience a musculoskeletal injury than their normal weight peers (BMI = 18.5-24.9 kg/m^2^) [16]. Obese firefighters also demonstrated significantly lower cardiorespiratory fitness and reduced strength compared to normal weight firefighters [3,17].

Firefighters suffer from high prevalence of cardiovascular disease (CVD)-related issues and CVD events are the leading cause of line-of-duty-death [1,18], accounting for almost half. This proportion of deaths is higher than for police (22%), other emergency medical service providers (11%) and on-the-job deaths for all occupations (15%) [18]. A number of studies examined obesity-related CVD risk factors among firefighters and found that obese firefighters are more likely to suffer from arterial stiffness, hypertension, low HDL cholesterol, high LDL cholesterol, high triglycerides, and more frequent fatal cardiac events [1,3,11,17,19-21].

Behavioral health issues, such as tobacco and alcohol use, also are concerns for the US Fire Service. While smoking prevalence (unadjusted estimate = 13.6%) for male career firefighters tended to be lower than that for adult males in the US and males in comparable occupations [22], prevalence estimates for smokeless tobacco and dual use (i.e., smokeless tobacco and cigarettes) were two- to three-fold higher than those reported for adult males in the US [22,23]. Similarly, problem drinking patterns, such as heavy and binge drinking, are quite common among male firefighters, even though there are significant penalties for intoxication on or off duty. For example, Carey and associates [24] found that 56% and 14% reported binge drinking and hazardous drinking patterns (i.e., binge drinking ≥ 8 /month), respectively. More recently, Haddock et al. [24] reported high prevalence of binge drinking (56%) and driving while intoxicated (9%) in a population-based sample of male career firefighters.

The US Fire Service has noted that, in the past, their focus has primarily been on acquiring and maintaining equipment/apparatus rather than the health and fitness of firefighters and emergency medical service (EMS) personnel who use the equipment and respond to critical incidents across the US [25]. Therefore, it is not surprising that there are a limited number of firefighter and EMS health promotion programs that have been developed and empirically-tested. One of the most notable is the PHLAME Study, which was the only randomized prospective trial that tested two treatment modalities (individual vs. team-based) focusing on nutrition, physical activity, and maintaining healthy bodyweight compared to medical monitoring only [8]. At one year follow-up, all groups gained weight, but less was gained in the intervention groups. The interventions did not result in significant improvements in cardiorespiratory fitness levels or healthy physical activity behavior [8]. A quasi-experimental examination of injuries and workers compensation claims in two PHLAME study fire departments and two matched departments from the same region that did not participate in PHLAME demonstrated important reductions in reported injury claims [26]. Leffer et al. [27] implemented a physician-based health promotion program and reported significant injury rate reductions over the study period. Several small, uncontrolled, single group diet only programs [28,29] reported modest body composition improvements in short term (12-week) studies.

The Fire Service Joint Labor Management Wellness-Fitness Initiative, 3^rd^ Edition (WFI) [25] is a comprehensive health promotion program developed by the International Association of Fire Fighters (IAFF) and the International Association of Fire Chiefs (IAFC), the primary labor and management organizations in the US Fire Service and the principal firefighter health stakeholders. The primary goal of the WFI is clearly articulated in the document’s forward:

“The ultimate goal of the comprehensive *Fire Service Joint Labor Management Wellness*-*Fitness Initiative* is to improve the quality of life of all uniformed personnel. The project seeks to demonstrate the value of investing wellness resources for the duration of uniformed personnel’s careers in order to maintain fit, healthy, and capable fire fighters and EMS responders. An effective program will minimize the expenditures on lost work time, workers compensation, and disability.” (IAFF, 2008; pp. 4) [25].

The WFI [25] consists of 58 pages of primary content and demonstrates that fire service labor and leadership organizations prioritize medical evaluation and fitness programs (i.e., 24/38 [63%] of health content pages; 24/58 [41%] of all pages).

Despite the fact that the WFI was implemented in 1999, with the third edition published in 2008 [25], there have been no large-scale, comprehensive evaluations of the WFI or programs emphasizing WFI goals on firefighter health. The IAFF [25] commissioned an evaluation in a small number of fire departments examining the average number of occupational claims and days lost from work, total incurred costs, and costs per claim. The evaluation covered the seven year periods prior to (1991–1997) and after (1998–2004) WFI implementation in four departments compared to four non-WFI departments. WFI departments had significantly lower rises in average claims (percent change for time periods = 5% vs. 22%), fewer average lost work days (percent change for time periods = −28% vs. 55%), and smaller increases in average total costs (percent change for time periods = 3% vs. 58%) when compared to non-WFI departments [25].

While the data are promising with respect to documenting the benefits of firefighter health promotion programs, previous approaches have been limited by small sample sizes of firefighters and/or fire departments, little or no sampling from broad US regions, and statistical approaches that did not address the clustering of firefighters within departments, the use of fire departments as the unit of analysis, or using data aggregated at the department level with no links to individuals, the last of which precluded investigators from addressing both individual (e.g., age, marital, fitness level, pre-existing health conditions, etc.) and department (e.g., size/number of personnel, call volume, services provided, setting, etc.) confounding factors that may contribute to the differences reported.

The purpose of this study was to compare purposively sampled fire departments from across the US that implemented important health promotion measures consistent with the WFI (note that less than 30% of departments appear to engage in any regular fitness programming or testing [11,26]) with those who have not on important fire service health measures. Because the medical and fitness sections of the WFI are the most prominent, we selected and compared 10 departments that had medical and fitness programs similar to that promoted by the WFI (i.e., a Wellness Approach; WA) with 10 departments that did not (i.e., Standard departments) have health promotion programs addressing these issues. Primary outcomes included measures of body composition, physical activity, general and behavioral health conditions, and factors related to the department culture. This approach builds on the nascent body of literature supporting the benefits of health promotion programs for firefighters [8,25-27] by: 1) using a large national sample of career fire departments that varied in size and mission to improve generalizability to the US Fire Service; 2) assessing a broad array of important individual firefighter health outcomes; and 3) addressing significant statistical limitations unaccounted for in previous studies because of data clustering when departments are the unit of selection or assignment [25,26].

## Methods

The data reported are from the baseline evaluation of an ongoing longitudinal cohort conducted by investigators from the Center for Fire, Rescue, and EMS Health Research at the National Development and Research Institutes (NDRI) and the University of Texas Houston Health Sciences Center (UTHHSC). The study was funded by the Assistance to Firefighters Grants program managed by the Federal Emergency Management Agency (FEMA) in the Department of Homeland Security (EMW-2009-FP-01971) and focused on the impact of wellness and fitness programs on health and safety in career firefighters. The protocol for the protection of human subjects for this study was approved by the NDRI and UTHHSC Institutional Review Boards.

### Department selection

A number of research strategies were considered and ruled out given their lack of feasibility when considering the funding mechanism’s time and funding constraints. First, a true experimental clinical trial was ruled-out because it would not be possible to randomly assign fire departments to implement the WFI, because it would require substantial funds and training (e.g., peer fitness trainers, medical evaluations) for even minimally acceptable execution. Second, a quasi-experimental approach involving random selection of WA and standard departments also was rejected because there is not an accepted comprehensive listing of fire departments or their status with respect to implementing WFI components. Therefore, we selected a purposive sampling approach of heterogeneous instances (i.e., extreme groups; [30]), which involved soliciting departments that implemented key WFI components (WA departments) and comparing them with departments matched with respect to size, call volume, staffing, and catchment area that did not implement key WFI components (Standard departments). We also worked to ensure that we recruited similar numbers of firefighters within the matched departments.

We used three methods to generate a pool of potential WA departments. First, we developed a list of departments that exemplified a strong commitment to health and fitness from the personal experience of researchers who work in the fire service. Second, our Expert Fire Service Panel (EFSP), composed of firefighters, fire chiefs, and other fire service-related personnel, generated a list of departments they believed best met the goals of the WFI. Third, we solicited participation through the listserv http://www.firefighterclosecalls.com, which was initiated in 1998 and is subscribed to by over 300,000 members and has over three million direct “hits” per month. These three steps resulted in a list of approximately 40 potential WA departments for screening. Based on our previous research conducted at departments across the US and a limited number of studies examining firefighter fitness and health promotion [11], it was apparent the career fire service is divided into a small proportion of departments generally following many WFI guidelines and a much larger proportion only following a limited number or none at all. In coordination with our EFSP, representatives of national organizations, and active Fire Chiefs and firefighters, we developed a formal screening instrument to narrow the pool of potential WA departments to a final list best reflecting WFI practices. We emphasized the WFIs medical and fitness criteria for screening departments because they represent the bulk of the total and health content in the WFI [25] and have been identified in the literature and by our EFSP as important factors in improving fitness, body composition, and operational readiness. The operationally-defined components included whether the department provided, at the time of screening:

1. NFPA 1582 [31] compliant annual medical physical examinations to all fire service personnel;

2. A designated health/fitness coordinator (this could be a professional position or filled by a firefighter as an extra duty);

3. Peer fitness trainers (PFTs) who matriculated in an approved program for the designation of PFT; and

4. Time for physical training/working out while on-duty for all fire service personnel.

Using our initial pool of potential WA departments, we contacted each department chief or designee and conducted a formal telephone screening to assess compliance with the above criteria. Department level data characteristics (e.g., department size, call volume, etc.) also were collected. Using all of the above information, we rated potential WA departments and narrowed our list to those 10 that best demonstrated implementation and reflection of the WFI aspirations. After selecting the initial 10 WA departments we sought EFSP and FEMA approval to finalize our WA selections.

Once our 10 WA fire departments were selected, we solicited Standard departments through the firefighter close calls listserve. We developed a comprehensive list of potential Standard departments that responded to the solicitation and matched them to WA departments using criteria such as approximate department size, geographic region, personnel composition, tenure in the fire service, and call volumes. EFSP also reviewed the selections for how closely they matched the WA departments. Each matched Standard department was telephoned to ensure that they did not meet any of the four key WFI medical and fitness criteria at the time of screening and if they did, we randomly selected another department. Next, we evaluated “face comparability” of WA and Standard departments by having our EFSP and FEMA consultants examine department characteristics and approve the lists of matched Standard departments.

Once both lists were finalized, we randomly selected three fire stations from each department; however in the larger departments we first randomly selected districts and then stations. These stations were then visited to recruit individual participants. For those departments with three stations or less, we conducted full census sampling. We oversampled participants in the four very large, metropolitan departments selected, two in the WA group and two in the Standard group, which was recommended by the EFSP and FEMA. The 20 departments represented a broad range with respect to size and number of personnel, with some large metropolitan departments having several thousand firefighters and more than one hundred stations to departments with less than 50 firefighters and only one station. In addition, we covered 14 US states, commonwealths, and/or territories and had at least one department in each of the four major US Census Bureau Regions.

While this approach cannot fully protect against potential selection bias, we implemented a number of strategies to minimize it given the quasi-experimental study design. First, we clearly and operationally-defined WFI criteria for the WA departments and we worked with the EFSP to ensure significant diversity in the WA departments with respect to region, department size and call volume. Second, we confirmed with department leadership that the WA departments met all operationally defined inclusion criteria and that the programs had been in existence for sufficient duration. For example, among the WA departments, the medical programs had been in place from 4–25 years and the fitness programs in place from 4–40 years. Third, we selected Standard departments that both we and our EFSP and FEMA advisors believed were most closely matched to each WA department and screened them to ensure they did not meet any of the WA inclusion criteria. Fourth, we included covariates in our statistical models to represent any measured differences in department or firefighter characteristics potentially influential to measured outcomes. These methods should ensure unusually strong comparability between our WA and Standard departments.

### Measures

Demographics (e.g., age, marital status, educational level, etc.), occupational history (e.g., current rank and position, years in the fire service, etc.), and self-reported health and medical history (e.g., history of heart disease, diabetes, hypertension, etc.) were collected along with the following:

#### Body composition

Height was assessed with a portable stadiometer. Body weight and BF% were determined using the Tanita 300, which is a digital scale with bioelectrical impedance. The Tanita 300 demonstrates strong concurrent validity when compared to the “gold standard,” Dual-Energy X-ray Absorptiometry (DEXA; r = 0.94; p < 0.001) [32]) for BF% estimation. It is a commonly used field measure because it is portable and accurate for estimating BF% [3,10]. WC was assessed using a spring-loaded tape measure in accordance with standard guidelines [33]. Obesity status was computed using BMI, BF%, and WC using standard cutpoints [33]. Self-perceptions about bodyweight also were assessed [34].

#### Physical activity, estimated VO_2_max, and exercise ratings

The Self Report of Physical Activity (SRPA) questionnaire [35] provides a global self-rating of physical activity patterns ranging from 0 (Avoids waking or exertion) to 7 (engages in >3 hours/week of heavy physical activity such as running, swimming, rowing, etc.) over the previous month. The SRPA’s validity compared to maximal oxygen consumption has been established [35].

Subjects’ age, gender, BMI, and SRPA score were used in a non-exercise model to estimate VO_2max_[35-40]. This method has been favorably compared with measured VO_2max_ and demonstrated equal, if not better accuracy than methods using sub-maximal exercise heart rate [35-40]. We selected this approach for its accuracy and feasibility, given the difficult nature of the field data collection process and cost limitations which precluded having firefighters report to a laboratory for a “gold standard” assessment. Aerobic capacity sufficient to exceed the NFPA minimum post-cardiac event exercise tolerance threshold was evaluated by using estimated VO_2max_ values to compute the suggested cutpoint of ≥12 METs (≈VO_2max_ ≥ 42ml/kg/min) [3,11,31].

We also asked firefighters to rate their on-duty physical activity frequency with the question “Most weeks, I exercise at the fire station or at work” with response options including never, some days, most days, and every day and duration by asking “Most times that I do cardio or aerobic exercise (e.g., jogging, brisk walking, bike, treadmill, etc.), I do an average of…each session”) or “Most times that I lift weights or do strength training, I do an average of ….each session” with response options ranging from none to >60 minutes [12].

#### General and behavioral health conditions

Participants were asked to rate their future risk of serious disease on a 5-point scale with anchors of “Not at all likely” and “Very likely” and the number of days during the past 30 that their physical health was not good. Measures similar to these have been used in studies of self-rated health in related occupations [41,42].

Blood pressure was measured digitally with the Omron HEM-711AC in accordance with epidemiological protocols [42]. The frequency on any injury (e.g., musculoskeletal strains or sprains, dislocations, lacerations, burns, etc.) during the past six months that was reported to Workers’ Compensation was assessed using a question adapted from the National Institute of Standards and Technology [43], and has been used in previous firefighter studies [15,16].

Firefighters were asked to self-report whether they ever had a physician diagnosed depressive or anxiety disorder. The Center for Epidemiological Studies Short Depression Scale (CES-D 10) was used to assess current depressive symptoms. The CES-D 10 is highly reliable in the general population (Spearman-Brown, split halves r = 0.85) and in patient samples (r = 0.90) [44]. Firefighters also were asked to rate their work stress and how much it interfered with work performance during the last six months [45]. Tobacco and alcohol use were assessed using questions from established epidemiologic surveys and used in previous studies [22,24,46].

#### Fire service culture

Firefighters were asked to indicate their level of job satisfaction on the following items: 1) “I am optimistic about my future success with this fire department”; 2) “I am satisfied with my job at the fire department”; 3) “I am satisfied with the morale of the people I work with in the fire service”; 4) “I am satisfied with the morale of the fire department”; and 5) “My work in the fire department gives me a sense of accomplishment.” Response options were a five-point Likert scale ranging from “Very much disagree” to “Very much agree” and scored in a continuous fashion, consistent with other similar scales [47]. In addition, we asked them to rate their perceptions about the availability and quality of exercise equipment provided by the department [12].

### Procedures

A core team of investigators traveled to each of the 20 fire departments for 3–8 days depending on the size and shift structure of the department (e.g., 24-hour vs. 48 hour, number of shifts staffed, etc.), thus maximizing the potential to recruit study participants from all available firefighters. Study travel dates were approved by each department’s leadership and/or points of contact. Firefighters who agreed to participate in the study were provided an overview of the study, the specific aims, risks, and benefits involved in study participation. However, we were unable to solicit firefighters who were on sick leave, vacation, or attending lengthy emergency calls during the study visits. Of the firefighters present and solicited during each baseline visit, 94.4% overall (N = 1,035) agreed to participate in the study and were consented. They were provided a survey to complete and moved through different assessment stations for physical examination.

### Statistical approach

Because of the small proportion of female firefighters (3.7%) in the US Fire Service [48] and in this study (n = 33; 3.2%) and our resulting inability to examine potential moderating effects of gender, only male firefighters (n = 1,002) were included in the study and statistical models. We examined differences between firefighters in WA and Standard departments across all demographic, fire service, and outcome domains (e.g., body composition, fitness and exercise, general and behavioral health, and fire service culture variables) and report percentages (discrete variables) and means (continuous variables) for variables in each domain. Table 1 provides the demographic and fire service characteristics of enrolled male firefighters and also summarizes department features.

**Table 1:** Firefighter demographics and work status by WA and standard department status

**Firefighter demographics**	**WA**^§^**(n = 522; 52**.**1)**	**Standard (n = 480; 47**.**9)**	**p-value**
Age (years)	39.9(8.5)	38.4(9.1)	0.006
Race/Ethnicity (%)			<0.001
-White, non-Hispanic	67.4	67.2	
-Black, non-Hispanic	2.9	6.1	
-Asian/Pacific Islander, non-Hispanic	14.4	5.9	
-Other, non-Hispanic	5.1	4.8	
-Hispanic, any race	10.3	16.1	
Marital status (% married or part of unmarried couple)	78.4	75.5	0.289
Served in military (% yes)	19.8	20.6	0.753
Education (%)			0.306
-Less than high school graduate	0.2	1.1	
-High school graduate or GED	9.3	8.6	
-Some college	68.0	69.6	
-College graduate or higher	22.5	20.7	
**Firefighter fire service status**			
Years in the fire service (years)	14.6(8.3)	13.6(8.9)	0.140
Total annual income (%)			0.004
-<$25,000	1.2	1.2	
-$25,000-$50,000	4.3	9.2	
-$50,001-$75,000	14.1	16.1	
-$75,001-$100,000	25.4	26.7	
->$100,000	55.0	43.8	
Rank (% company or chief officer)	22.6	30.0	0.010
Hours per week worked at department	59.0(13.1)	55.8(15.6)	0.001
Shift Type (%)			<0.001
–24	76.1	90.1	
–48	22.3	6.5	
-Other	1.6	3.4	
Call Type (%)			
-Fire	1.2	1.6	0.792
-EMS	0.6	0.9	
-Both	98.2	97.5	
Second Job Outside of Fire Department (% yes)	35.4	55.4	<0.001
**Department characteristics (N = 20)**	**WA (n = 10)**	**Standard (n = 10)**	
Size (n)			0.844
-Small	3	4	
-Medium	4	4	
-Large/Metro	3	2	
Setting (n)			0.706
-Rural	1	3	
-Suburban	6	4	
-Urban	1	1	
-Mixed Urban	2	2	
Region (n)			0.999
-Pacific/West	4	4	
-Mountain/Central	4	4	
-East/Southeast	2	2	

*§WA*=Wellness approach.

Firefighters in WA departments differed from those in Standard departments in age and a number of departmentally-oriented factors (e.g., fire service income and total income from all sources, department rank, hours worked in the department, shift type, and whether or not they had a second job).

Table 1 also summarizes department-level characteristics. Fire departments were classified into categories based on their size, setting/type, and region. With regard to department size categories, Large/Metro departments (n = 5 total) were those with more than 350 personnel based on the fire services’ typical classification [49]. Small departments (n = 7 total) were defined as those with three or fewer stations, and medium size departments (n = 8 total) were those with more than three stations but fewer than 350 personnel. Departments within large cities were classified as urban (n = 2 total). Those not in a large city but nearby one were considered suburban (n = 2 total). Departments not in or near a large city were considered rural (n = 4 total). In addition, some department were classified as mixed (n = 4 total) because they covered urban, suburban, and rural cities/towns. Finally, departments also were classified using broad US Census-based regions similar to those we used on our previous national study of firefighter health concerns [50,51]; the regions were Pacific/Western (n = 8 total), Mountain/Central (n = 8 total), and Northeastern/Southeastern (n = 4 total). As can be seen in Table 1, there were no significant differences between WA and Standard departments with regard to size, setting/type, or region.

All models were adjusted for firefighters’ age, race/ethnicity, total income, whether or not they had a job outside the department, and occupational rank. To account for the sampling approach of the study and variability among departments, we also included department as a random covariate in each statistical model. Thus, differences among departments (e.g., shift structure) are addressed through this random factor. For continuous outcomes, statistical models were developed using SAS 9.3 PROC MIXED. Models with discrete outcomes were constructed with SAS 9.3 PROC GLIMMIX and produced adjusted least squared means. For dichotomous and ordinal variables (e.g., rating scales) [52], the logit linking and cumulative logit linking functions within GLIMMIX were used. Statistical models of ordinal outcomes produced odds ratios which represent the effect of the predictor variables on the odds of being in a lower rather than a higher ordered category while the models for dichotomous outcomes represented the odds of having the outcome of interest (e.g., being obese, hypertensive, etc.) with WA department status as the reference group.

## Results

### Body composition and physical activity/exercise

Table 2 presents differences on the body composition and physical activity/exercise measures between WA and Standard departments.

**Table 2:** **Comparisons** (**Least square mean**±**SE or unadjusted** %*) **between WA and standard departments on body composition and physical activity**/exercise measures**

**Body domposition**	**WA**^§^**(n = 522; 52**.**1)**	**Standard (n = 480; 47**.**9)**	**p-value**
BMI (kg/m^2^)	28.4(0.5)	30.5(1.1)	0.011
BF% (%)	23.1(0.8)	26.9(1.7)	0.002
WC (cm)	96.2(1.2)	102.2(2.8)	0.006
Obesity status			
-BMI defined (% BMI ≥ 30.0)	25.3	35.6	0.002
-BF% defined (% BF% > 25%)	33.3	43.5	<0.001
-WC defined (% WC > 102.0 cm or 40.0 inches)	23.0	32.8	<0.001
Weight self-perception (%)^¥^			0.016
-Underweight	6.0	4.1	
-About the right weight	35.0	31.8	
-Overweight	59.0	64.2	
**Physical activity and exercise**			
Physical activity level (SRPA)	5.0(0.2)	4.2(0.5)	0.022
Estimated VO_2max_ (mL/kg/min)	40.7(0.6)	37.5(1.3)	0.001
Met NFPA Standard (12.0 METs; % yes)	46.8	43.0	<0.001
Exercise at fire station (%)			<0.001
-Never	4.4	19.8	
-Some days	22.7	39.7	
-Most days	43.9	27.7	
-Everyday	29.0	12.9	
Most times I do aerobic exercise (%>30 minutes/session)	62.3	59.2	0.261
Most times I do strength training (%>30 minutes/session)	58.8	51.3	0.029

*Adjusted mixed models for categorical or ordinal outcomes with adjusted ORs [AOR) presented in text.

**All models were adjusted for firefighters’ age, race/ethnicity, total income, whether or not they had a job outside the department, occupational rank, and differences due to department variability.

*§WA*=Wellness approach.

^¥^Variable dichotomized in the generalized linear mixed model to “about the right weight” vs. under- and overweight combined.

Firefighters in WA departments demonstrated superior body composition regardless of method and were significantly less likely to be obese when using definitions based on BMI, WC, or BF% (see Table 2 for adjusted Least Square Means for BMI, BF%, and WC). For example, firefighters in WA departments were 42% (adjusted [A]OR=0.58; 95% CI=0.41-0.82), 68% (AOR=0.33; 95% CI=0.18-0.59), and 46% (AOR=0.54; 95% CI=0.39-0.73) less likely to be obese based on BMI, WC, and BF% standards, respectively, when compared to firefighters in Standard departments. It also is notable that only Standard departments had firefighters meeting the criteria for Class III obesity (BMI ≥ 40 kg/m^2^; n =16 or 3.4% vs. 0.0% for the WA departments), also referred to as “clinically severe obesity” which generally is when individuals are considered candidates for bariatric surgery [32]. Finally, firefighters in WA departments were 36% (AOR = 0.64; 95% CI = 0.45-0.92) less likely to perceive their weight status as being under- or overweight than those in Standard departments (see Table 2).

WA firefighters had significantly higher levels self-reported physical activity and estimated VO_2max_ (see Table 2). WA firefighters were five time more likely (AOR = 5.19; 95% CI = 2.49-10.83) to meet the NFPA minimum post-cardiac event exercise tolerance threshold [7,11,31] and 40% (AOR = 1.40; 95% CI = 1.04-1.89), more likely to report engaging in more than 30 minutes per session of strength training, and they were 74% (AOR = 0.26; 95% CI = 0.20-0.35) less likely to be clustered in the lower categories of exercise frequency at the station than their counterparts in Standard departments.

### General and behavioral health conditions

Table 3 presents the differences between WA and Standard departments on the various indicators of general and behavioral health.

**Table 3:** **Comparisons** (**Adjusted least square mean**±**SE or unadjusted** %*) between WA and Standard departments on general and behavioral health conditions and substance use**

**General and health conditions**	**WA**^§^**(n = 522; 52**.**1)**	**Standard (n = 480; 47**.**9)**	**p-value**
Self-rating of risk for serious disease in the future	2.8(0.4)	3.2(0.1)	<0.001
Number of days in poor health (during last 30; days)	3.3(0.92)	3.5(0.4)	0.717
Self-reported health conditions			
-Type 2 diabetes (% yes)	1.0	2.0	0.912
-High blood pressure (% yes)	11.8	14.4	0.324
-High cholesterol (% yes)	27.9	26.0	0.167
-Heart disease (% yes)	1.2	1.1	0.837
-Stroke (% yes)	0.2	0.0	0.650
-COPD (% yes)	0.2	0.2	0.979
-Sleep apnea (% yes)	5.6	5.1	0.831
-Arthritis (% yes)	4.8	6.0	0.804
Hypertension (measured at station; % SBP ≥ 140 and/or DBP ≥ 90)	16.2	23.7	0.001
Any injuries reported to workers compensation in last 6 months (% yes)	11.4	7.0	0.032
**Behavioral health conditions**			
Physician diagnosis of anxiety disorder (% yes)	3.2	10.7	<0.001
Physician diagnosis of depressive disorder (% yes)	6.2	6.8	0.231
Depressive symptom score (CESD10)	1.7(0.1)	1.8(0.1)	0.407
Stress at work while carrying out duties during last 6 months	1.8(0.1)	1.8(0. 3)	0.898
Stress at work interfered with performing duties during last 6 months	0.5(0.1)	0.7(0.2)	0.449
Smoking status (% Current)	4.6	13.9	<0.001
Smokeless tobacco user (% Current)	13.4	12.5	0.906
Alcohol consumption past 30 days (number drinks x days drank)	35.4(3.2)	38.2(3.1)	0.476
Alcohol use category (%)^¥^			0.063
-Abstinent	16.9	21.1	
-Moderate; 1–2 drinks per day	43.7	32.8	
-Heavy; 3 or more drinks per day	39.5	46.1	
Binge drinker (% > 5 drinks in one occasion)	43.9	50.8	0.058

*Adjusted mixed models for categorical or ordinal outcomes with adjusted ORs [AOR) presented in text.

**All models were adjusted for firefighters’ age, race/ethnicity, total income, whether or not they had a job outside the department, occupational rank, and differences due to department variability.

*§WA*=Wellness approach.

^¥^Variable dichotomized in the generalized linear mixed model to “heavy drinker” vs. moderate and abstinent combined.

Firefighters in WA departments had significantly lower ratings for risk of future serious illness (see Table 3) and they were almost 50% (AOR = 0.51; 95% CI = 0.35-0.76) less likely to meet the definition of hypertensive at the time of the evaluation when compared to their colleagues in Standard departments. Injuries of sufficient severity to report to workers compensation (i.e., 11.4% vs. 7.0% for WA and Standard, respectively), were significantly higher among firefighters in WA departments (AOR = 1.74; 95% CI = 1.05-2.90) when compared to those in Standard departments.

With respect to behavioral health factors, firefighters in WA departments were significantly less likely to report being diagnosed with an anxiety disorder (AOR = 0.27; 95% CI = 0.14-0.52) or to currently smoke (AOR = 0.30; 95% CI = 0.17-0.54) when compared to firefighters in Standard departments. No other behavioral health variables significantly differed between firefighters in WA and Standard departments.

### Department culture

Firefighters in WA departments were consistently more likely to report greater optimism, job satisfaction, satisfaction with their department and colleagues, and a greater sense of accomplishment than firefighters in Standard departments (see Table 4).

**Table 4:** **Comparisons** (**Least square mean** ±** SE or unadjusted** %*) between WA and standard departments on department culture**

**Fire department culture**	**WA**^§^**(n = 522; 52**.**1)**	**Standard (n = 480; 47**.**9)**	**p-value**
Job satisfaction			
-Optimistic	4.1(0.1)	3.7(0.2)	0.033
-Satisfied with job	4.2(0.1)	3.8(0.2)	0.001
-Satisfied with the morale of co-workers	3.6(0.1)	2.9(0.2)	<0.001
-Satisfied with morale of the fire department	3.6(0.1)	3.1(0.2)	<0.001
-Sense of accomplishment	4.2(0.1)	3.9(0.2)	0.012
Resistance training equipment availability (% yes)	97.4	93.0	0.566
Endurance equipment availability (% yes)	95.8	87.6	0.361
Team sports equipment availability (% yes)	30.7	7.9	<0.001
Exercise equipment rating	3.8(0.1)	3.8(0.3)	0.957

*Adjusted mixed models for categorical or ordinal outcomes with adjusted ORs [AOR) presented in text.

** All models were adjusted for firefighters’ age, race/ethnicity, total income, whether or not they had a job outside the department, occupational rank, and differences due to department variability.

*§WA*=Wellness approach.

Firefighters in WA departments were significantly more likely to report greater availability of team sports equipment (AOR = 92.9; 95% CI = 5.18-999.9) than those in WA departments, but the width of the confidence interval suggests that the AOR is not very stable. There were no statistical differences between firefighters in WA and Standard departments with respect to the perceived availability of endurance or strength training equipment and their overall ratings of their departments’ exercise equipment.

## Discussion

Compared to firefighters from Standard Departments, those from WA departments were leaner, less likely to be obese (on any body composition measure) or have hypertension, more physically active, had greater estimated cardiorespiratory endurance, rated themselves as healthier, were less anxious, and were significantly less likely to smoke. In addition, they had greater morale and satisfaction with their jobs, colleagues, and departments. This study provides strong evidence for an association between whether a fire department has a well-developed health promotion program and the health and wellness of firefighters. Although it is not possible to make causal attributions about this association given the selection process used to allocate departments to the WA and Standard conditions, it is consistent with a number of large systematic reviews which have documented beneficial effects of comprehensive health promotion programs on improving physical activity behavior and reducing BMI/weight and smoking [53-55].

Obesity prevalence was high in Standard departments, regardless of body composition method and the estimates were similar to the US general adult male population [4] and estimates from firefighters in previous population-based studies [3]. Even more striking was the fact that 3.4% of firefighters in standard departments were Class III obese (BMI≥40.0 kg/m^2^), also closely paralleling adult males in the general US population [4]. Given the strenuous physical demands of fire suppression, rescue, and emergency medical tasks, this is a significant issue for the fire service and substantially increases risks for morbidity and mortality [1]. For example, obesity in firefighters has been associated with more CVD risk factors (e.g., arterial stiffness, hypertension), more frequent fatal cardiac events, and greater risk of incident musculoskeletal injuries, injury-related absenteeism, and disability [1,3,9-11,16,17,19-21]. Not surprising, experts have called for work restrictions and medical evaluations on firefighters with Class III obesity until they lose weight or improve their fitness [1].

Firefighters in WA departments were significantly leaner and demonstrated obesity prevalence between 9.8-10.3 percentage points less than those in Standard departments on all body composition indices. None of the WA department firefighters were Class III obese and they were more physically active on all relevant measures. This suggests that firefighters in WA departments would be more likely to meet requirements for operational readiness and less likely to suffer from all of the conditions linked to obesity among firefighters [1,3,9-11,16,17,19-21]. These findings can only be inferred because this study was cross-sectional and longer-term outcomes were not available for this report.

It was notable that firefighters’ self-perception of their weight was quite different from their actual status. For example, we found that nearly 32% of Standard department firefighters and 35% of WA firefighters perceived their weight as “about right”, yet only 16.6% and 18.1%, respectively, actually met the definition for normal weight status (BMI = 18.5-24.9 kg/m^2^). This is even more remarkable for firefighters in Standard departments given that the prevalence of obesity using any body composition standard was significantly higher than found among WA firefighters. Kay and colleagues [56] reported that the majority of the firefighters in their survey (53%) rated their weight as “about right” and only 44% considered themselves overweight, even though nearly 85% were actually overweight or obese (BMI ≥ 25 kg/m^2^). Similarly, Baur and associates [57] reported that only 8% of obese (37% of the sample) and 32% of overweight (51% of the sample) male firefighters (N = 768) correctly classified themselves as obese or overweight. Regular and targeted surveillance may serve the purpose of confronting this potential misperception and motivate some firefighters to change weight-related health behaviors [7].

Injury prevalence based on reports of workers’ compensation claims was higher among firefighters from WA versus Standard departments, which represents a deviation from longitudinal evaluations of the WFI and the PHLAME firefighter health promotion interventions [25,26]. However, we selected WA departments based on just the medical and fitness components of the WFI, thus omitting one of the other cores, albeit less emphasized, areas of injury rehabilitation [25]. Nevertheless, the proportion of injuries resulting in a workers’ compensation claim were low for both groups and we did not assess in what activity context the reported injuries occurred. In our previous cohort study of injury risk [15,16], we found that more active firefighters had lower prevalence of non-exercise injuries, but were more likely to be injured while exercising. It is possible that firefighters in the WA departments reported more injuries because they were more physically active and more likely to engage in strength training [15,16]. In addition, it is possible that their departments encourage such reporting or that they felt more comfortable about reporting injuries.

Regardless, it is likely that the bulk of costs associated with firefighter injuries and their treatment and rehabilitation can be attributed to non-exercise injuries for the following reasons: 1) most exercise-related injuries (85.2%) reported by firefighters are sprains and strains [14], of which 100% were rated as minor; 2) exercise-related injuries among firefighters tend to represent less than one-third of all injuries [14,15]; 3) firefighters who are more active are considerably less likely to be injured in non-exercise-related activities such as fire suppression, rescue, and emergency medical tasks [15]; and 4) lack of fitness is the second-leading contributing factor to line-of-duty injuries [58]. Longitudinal studies have demonstrated that when injury prevention and rehabilitation are emphasized in firefighter health promotion programs, reductions in injuries and related costs are a likely outcome [25,26].

Behavioral health outcomes have not been previously been examined in firefighter health promotion studies [25,26]. Firefighters in WA departments were significantly less likely to have been diagnosed with an anxiety disorder or currently smoke cigarettes. However, it should be noted that even among firefighters in the Standard departments, current smoking prevalence was below men in the general US population and consistent with the low estimates previously documented among firefighters in a population-based study [22]. There are several factors that likely have influenced firefighters in both WA and Standard departments to smoke less including “no smoking contracts” as a condition of employment, disease presumption and indoor smoking laws, the negative impact of smoking on fitness, and firefighters witnessing the detrimental effects of smoking on the health of the people they serve as well as its role in household fires [51].

At least two observations may help explain the extremely low smoking prevalence among WA firefighters. First, firefighters in WA departments were more physically active and exercised more often than those in Standard departments, suggesting lifestyles less compatible with smoking. Second, the higher prevalence of anxiety disorders among firefighters in Standard departments also might have influenced the higher proportion of smoking or vice versa, given the cross-sectional nature of the study. For example, Moylan et al. [59] documented consistent associations between smoking and anxiety in a systematic review. Haddock et al. [22] also documented a strong cross-sectional association between smoking and diagnosis of an anxiety disorder in a population-based study of male career firefighters, noting that current smokers were more than five times more likely (OR = 5.8; p = 0.010) to have been diagnosed with an anxiety disorder when compared with never smokers. However, it is unclear why firefighters in Standard Departments were so much more likely to have been diagnosed with an anxiety disorder. It is possible that this association is a result of the clustering of poor health indicators among firefighters in Standard departments, i.e., they were more obese, more likely to smoke, and less physically active, all things associated with anxiety [60,61]. There were no significant group differences in reports of job-related stress or other mood disorders. In addition, it is unclear why departments without wellness programs might be more likely to attract firefighters with anxiety disorders (using a self-selection argument), so this finding is puzzling and difficult to explain.

Current smokeless tobacco use was similar between firefighters in WA and Standard departments. The prevalence was substantially higher than the general male adult US population and the highest documented among any occupational groups [60], but consistent with previous population-based studies reporting high smokeless tobacco prevalence among male firefighters [22,23]. Reasons for high prevalence of smokeless tobacco use in the fire service overlap substantially with the reasons documented above for reductions in cigarette smoking, along with several other factors: 1) smokeless tobacco use can be concealed easier than smoking even in the context of “no tobacco use contracts”; and 2) smokeless tobacco is sometimes viewed as cheaper than smoking, and is arguably less likely to impact operational readiness and health status [22,23,51]. Greater likelihood of smokeless tobacco use in male firefighters also tends to be associated with problematic alcohol use behaviors [62], another critical behavioral health issue among male career firefighters [24,63].

Binge and heavy drinking were common among both groups, but firefighters in Standard departments were more likely to be heavy drinkers. The high prevalence of binge drinking among both groups are consistent with previous studies that have reported binge drinking prevalence estimates of 56% in both a study of male firefighters from one Northeastern department [63] and a population-based study of male career firefighters, respectively [24]. The overall prevalence of binge drinking in our sample was more than double that of adult males in the US, suggesting that firefighters in both WA and Standard departments are at increased risk for the consequences of binge drinking [64]. The WFI [25] highlighted excessive alcohol use as an important health issue that needs greater attention in the fire service, particularly because the binge drinking behaviors are likely compressed into fewer days per month given the typical firefighter work schedules and department policies that do not allow drinking while on duty or for varying time periods before shifts [65].

Study strengths include that this is the first study to compare fire departments with strong wellness programs to those without wellness programs on a number of WFI-relevant health outcomes using a large, national sample of firefighters and fire departments varying in size, call volume, and geographic location. In addition, it is the first study to use statistical methods appropriate for sampling departments rather than individuals. However, our study is limited because of its cross-sectional design, thus restricting our ability to make causal inferences about the direction of the relationships between having health promotion programs and health outcomes. For example, our WA departments could have healthier firefighters only because they are more likely to attract, hire, and retain firefighters who are more interested in health and wellness rather than their better health being attributable to departments having such programs. Ultimately, only well-designed randomized or quasi-experimental trials can best address the effectiveness of health promotion programs for the fire service, and our findings strongly support the need for such research to confirm the benefits health and wellness programs in an appropriately powered sample of fire departments in the US. Also, although we compare WA and Standard Departments on a wide variety of health outcomes, some potentially important outcomes such as dietary intake, illicit drug use, and quality of sleep were not included in comparisons. Thus, future studies should examine the impact of health promotion programs on other aspects of firefighter health and readiness.

## Conclusion

Firefighters in departments purposively selected based on having strong wellness programs (WA) were healthier along a number of health dimensions including having better overall body composition, lower prevalence of obesity, anxiety disorders, and smoking, and greater levels of physical activity/exercise, and job satisfaction. The benefits of firefighter health promotion programs have been documented previously in smaller and regionally-restricted prospective studies examining a limited number of health parameters, such as showing that health promotion programs focused on injury prevention and rehabilitation resulted in lower costs associated with injury-related workers compensation claims [8,25,26]. In addition, worksite health promotion programs have generally demonstrated beneficial effects with respect many of the health issues prioritized by the US Fire Service including body composition, physical activity, and tobacco and alcohol use [53-55].

Our study adds to the body of evidence by demonstrating that firefighters in departments with well-developed health promotion programs that were consistent with the goals of the WFI [25] and also those suggested the American Heart Association, for comprehensive worksite health promotion programs [66], were healthier and have higher operational readiness when compared to firefighters in departments without such programs. For example, Finkelstein and colleagues [67] documented the considerable incremental health care costs associated with extreme (Class III; BMI ≥ 40.0 kg/m^2^) obesity, so the fact that even 3.4% of the firefighters in the Standard departments met this criterion suggest that Standard departments would have higher healthcare costs associated with these individuals and lower operational readiness. Prospective cluster-randomized trials or quasi-experimental studies of firefighter health promotion programs are needed to more clearly document the benefits of wellness programs.

Our study also identified and/or replicated areas in need of improvement. For example, regardless of department status and the fact that WA firefighters were more active, the majority of firefighters in both groups did not meet the NFPA minimum post-cardiac event exercise tolerance threshold [7,11,31]. Also, both groups had very high prevalence of smokeless tobacco use and binge drinking/heavy drinking, estimates that were consistent with data from previous population-based studies, suggesting that more emphasis on these behavioral health issues is desperately needed in the fire service.

## Competing interests

All authors declare that they have no competing interests.

## Authors’ contributions

WSC P was involved in the study origination and design, obtaining funding, data analysis and interpretation, and was chiefly responsible for writing the manuscript. CK H was involved in the study origination and design, obtaining funding, data analysis and interpretation, and critical revision of the manuscript. SA J was involved in the study origination and design, study supervision, data acquisition, and critical revision of the manuscript. N J was involved in study design, interpretation of results, and critical revision of the manuscript. RS D involved in study origination and design, obtaining funding, interpretation of results, and critical revision of the manuscript. All authors read and approved the final manuscript.

## Pre-publication history

The pre-publication history for this paper can be accessed here:

http://www.biomedcentral.com/1471-2458/13/805/prepub
